# Quality Assessment of Health Information on Social Media During a Public Health Crisis: Infodemiology Study

**DOI:** 10.2196/70756

**Published:** 2025-10-24

**Authors:** Rozita Haghighi, Mohsen Farhadloo

**Affiliations:** 1 Department of Supply Chain and Business Technology Management, John Molson School of Business, Concordia University, 1455 De Maisonneuve Blvd, W, MB 12.359, Montreal, QC, H3G 1M8, Canada, 1 514-848-2424

**Keywords:** health information, quality assessment, JAMA benchmarks, DISCERN, infodemic, health crisis, public health, Journal of the American Medical Association

## Abstract

**Background:**

The quality of health information on social media is a major concern, especially during the early stages of public health crises. While the quality of the results of the popular search engines related to particular diseases has been analyzed in the literature, the quality of health-related information on social media, such as X (formerly Twitter), during the early stages of a public health crisis has not been addressed.

**Objective:**

This study aims to evaluate the quality of health-related information on social media during the early stages of a public health crisis.

**Methods:**

A cross-sectional analysis was conducted on health-related tweets in the early stages of the most recent public health crisis (the COVID-19 pandemic). The study analyzed the top 100 websites that were most frequently retweeted in the early stages of the crisis, categorizing them by content type, website affiliation, and exclusivity. Quality and reliability were assessed using the DISCERN and JAMA (Journal of the American Medical Association) benchmarks.

**Results:**

Our analyses showed that 95% (95/100) of the websites met only 2 of the 4 JAMA quality criteria. DISCERN scores revealed that 81% (81/100) of the websites were evaluated as low scores, and only 11% (11/100) of the websites were evaluated as high scores. The analysis revealed significant disparities in the quality and reliability of health information across different website affiliations, content types, and exclusivity.

**Conclusions:**

This study highlights a significant issue with the quality, reliability, and transparency of online health-related information during a public health challenge. The extensive shortcomings observed across frequently shared websites on Twitter highlight the critical need for continuous evaluation and improvement of online health content during the early stages of future health crises. Without consistent oversight and improvement, we risk repeating the same shortcomings in future, potentially more challenging situations.

## Introduction

### Background

As the COVID-19 pandemic unfolded, the world faced unprecedented challenges, prompting urgent and restrictive responses globally. Governments across the globe responded with unprecedented measures, such as closing international borders, lockdowns, and travel restrictions [1,2]. These extensive restrictions disrupted global connectivity and daily routines [3].

Digital channels, including social media, are widely used for communication and community-building in noncrisis times, but their use was scaled and intensified during the COVID-19 pandemic [4], becoming critical for delivering timely updates, guidance, and a sense of connection amid enforced isolation, especially in limited-income and resource countries and remote areas, where they provide immediate access to vital health information and enhance patient autonomy [5,6]. The use of social media resources during the COVID-19 pandemic was driven by multiple concerns, including public interest in understanding the cause, illness, treatment, interventions, and circulating information, including misinformation and disinformation, leading many to seek health care information on social media [7-9]. The shift to digital platforms during the pandemic is double-edged [10]. While they efficiently disseminate critical information, potentially saving lives, they also open the door to a flood of false information [11]. In such critical times, where false information can spread like wildfire, the need for reliable and high-quality information is not just important—it is a matter of survival.

Recent research indicates that false information—including misinformation and disinformation (categorized by the author’s intent) [12-14]—spreads faster than facts on social media platforms, despite their potential for health education [15]. As the health crisis narratives evolve, distinguishing between fact and false information becomes increasingly difficult [10]. This leads to an “infodemic,” which describes the overwhelming spread of information, both accurate and misleading, across digital and traditional channels during a disease outbreak [16]. This includes both accurate scientific information and false narratives that spread across a range of traditional and new communication channels, including social media platforms [17].

Recognizing the serious impact of the infodemic, public health organizations, governments, and social media platforms initiated actions to combat misinformation, disinformation, and the infodemic [18]. In addition to enhancing fact-checking services, platform-level interventions, such as algorithmic adjustments, applying labels and warnings to questionable content to direct users to credible sources, and content amplification policies [10,15], different frameworks, such as Germany’s Social Listening and Integrated Analysis and Nigeria’s Social and Community Listening Systems, were developed, aligning with fact-checking, to monitor and address the COVID-19 infodemic and risk communication, leveraging real-time data and community insights to support effective public health responses. [19-21]

Ensuring access to reliable health information on digital platforms, such as social media microblogging networks, is a critical challenge during public health crises. During the COVID-19 pandemic, these platforms were key sources of health information [22], though health care providers remained the most trusted [23]. Reliable and trustworthy online health information supports informed health decisions and helps maintain public trust in digital health resources [24-26]. While we admit digital health information includes various sources such as search engines, peer communities, artificial intelligence assistants, and health apps, we mainly focus on social media platforms. Within this broader digital ecosystem, social media platforms, such as Twitter (subsequently rebranded X), function as a key amplification channel, facilitating the widespread circulation of health-related links from government, academic, medical, and news websites [27]. Therefore, assessing the quality of online health information is vital to address these challenges for future pandemics, such as COVID-19.

Ongoing research on analyzing the quality of health information on social media is abundant. Researchers have mainly analyzed the quality of retrieved information from popular search engines, such as Google (Google LLC), Yahoo (Yahoo Inc), and Bing (Microsoft Corporation) and concluded that a significant portion of health information retrieved is of poor quality, further emphasizing the need for enhanced quality assurance protocols across digital health platforms [28-30]. For instance, Cuan-Baltazar et al [31] aimed to assess the quality of online COVID-19 information by analyzing the first 110 results from a Google search. The findings indicated that a significant portion of the websites failed to meet basic quality criteria, highlighting the prevalence of poor-quality health information available to the public.

While research has shown that during health crises, public health agencies and government stakeholders across the globe use social media, such as Twitter, to disseminate information regarding situations, risks, and personal protective action inhibiting disease spread [32,33], the quality of health information on Twitter has not been addressed. To fill this gap and given the significance of the early phases of a health crisis, in this research, we aim to investigate the quality of top-circulated information on Twitter during the early stages of a public health crisis.

Our research specifically examines the quality of information on 100 websites that were most frequently retweeted during the early stages of the COVID-19 pandemic, in March 2020, a defining example of a global public health crisis. Such emergency moments are crucial for spreading accurate and timely information as the public looks for guidance and updates on the rapidly changing situation. We focused on key aspects, such as transparency, reliability, and overall quality of the information. To achieve this, we used the JAMA (Journal of the American Medical Association) and DISCERN tools to ensure a robust analysis. This comprehensive assessment covers the most important elements of evaluating online content, with minimum overlapping of these aspects.

By using these tools in our research, we aimed to provide a detailed and comprehensive evaluation of the quality of health information shared on Twitter. Our findings offer valuable insights into the state of health information on social media and highlight areas that need improvement during the early stages of future health crises. Ultimately, this will contribute to better public health communication and help combat false information for future public health emergencies.

### Literature Review

The quality of health-related content on social media is a significant concern due to the proliferation of digital platforms, leading to an unprecedented flow of health information accessible to a broad audience. In the health care domain, there are validated tools developed to assess the quality of health information considering aspects such as quality, reliability, transparency, readability, suitability, usability, design, and understandability. Various tools and frameworks, including DISCERN [34] and JAMA Benchmark [35], have been used for evaluations.

Researchers have used these tools to assess the quality of health information related to particular diseases, such as first metatarsophalangeal joint fusion [36], breast cancer [37], treatment of the mouth in systemic sclerosis [38], cervical cancer [29], type 2 diabetes [39], spontaneous coronary artery dissection [30], pancreatic cancer [28], inguinal hernia surgery [40], thyroid cancer [41], and COVID-19 [42].

Many studies have concentrated on evaluating the quality of health information on websites indexed by search engines, such as Google [43], Yahoo [44], Bing [45], and AOL (America Online) [46]. Most of these studies have generally concluded that the quality of health information related to various diseases retrieved by search engines was poor, lacking in reliability, transparency, and other key quality metrics.

In literature, other than websites, some scholars have focused on platforms that primarily offer video content to disseminate health information. The quality of health content found on these platforms, such as TikTok (ByteDance Ltd [47]), YouTube (Google LLC [48,49]), Bilibili (Bilibili Inc) [50], Douyin (ByteDance Ltd [51]), Weibo (Sina Corporation [52]), Facebook (Meta Platforms, Inc [53]), Instagram (Meta Platforms, Inc [54]), highlighting the attention given to content reliability, has also been evaluated in the literature.

Although the quality of video content found on social networking platforms, such as Facebook and Twitter, has been analyzed [53], to the best of our knowledge, the quality of health information found on these networks has not been studied. The rapid spread of potentially unreliable health information through these platforms presents unique challenges in ensuring the accuracy and reliability of content. This focus overlooks the unique dynamics of social media platforms, namely Twitter, in the early stages of a health crisis, where information dissemination is rapid and user-generated content plays a significant role. Therefore, a gap remains in the literature regarding the evaluation of health information quality specifically on Twitter, highlighting the need for targeted research in this area. Understanding and improving the quality of health information on microblogging networks, such as Twitter, is crucial for effective public health communication and combating false information during public health emergencies.

## Methods

### Overview

In this research endeavor, we evaluate websites involved in disseminating information related to the most recent public health crisis (COVID-19 pandemic) on the Twitter platform (subsequently rebranded X). Twitter, a leading social media platform, boasts over 450 million active users monthly and records approximately 6000 tweets per second. Unlike platforms that allow extensive posts, Twitter limits messages to 280 characters, promoting concise and precise communication. This characteristic makes Twitter a powerful tool for quickly spreading ideas on various topics, including politics, news, and global events.

#### Data Collection Process

We selected March 2020 as our study period, coinciding with the World Health Organization’s declaration of COVID-19 as a pandemic. Our data source was the “COVID-19 Tweets dataset” [55], which aggregates English tweets related to COVID-19 collected through the Twitter application programming interface. This dataset contains tweet IDs only, in accordance with Twitter’s data sharing policies. We therefore developed a Python (Python Software Foundation) script using the Twarc library to retrieve full tweet content, which we subsequently stored in a MongoDB database. Through this rehydration process, we obtained approximately 28.4 million tweets from March 2020.

#### Website Selection Process

To examine the information sources users encountered on Twitter, we identified tweets containing URLs and analyzed the most frequently shared links during our study period. We ranked websites by retweet frequency, reasoning that the most retweeted URLs represented the primary information sources users accessed while seeking COVID-19 information. Our analysis concentrated on the top 100 websites based on this ranking. Two researchers independently assessed health information quality across selected websites, resolving disagreements through discussion until consensus was reached.

#### Exclusion Criteria

We applied several exclusion criteria to ensure analytical focus. Websites offering primarily non-English content, statistical dashboards, video materials, or requiring paid access were removed from consideration.

Two independent researchers assessed the quality of health-related information on these websites, and any disagreement was resolved by consensus before the final analysis.

### Website Categorizations

We categorized the websites based on their affiliation, content type, and the focus of their topic and content. The websites were classified by their affiliation (commercial, news, university, medical center, nonprofit organization, or government), the type of content they provided (medical facts, clinical trials, human interest stories, and questions and answers), and the focus of their content (whether the site was exclusively dedicated to COVID-19 or if COVID-19 content was only a part of the site).

### Evaluation Frameworks

We applied established evaluation frameworks to assess the transparency, quality, and reliability of the health information on these websites. We used the JAMA benchmarks and DISCERN assessment tools to critically evaluate each website’s content. The JAMA benchmarks focus on authorship, attribution, disclosure, and currency, while the DISCERN instrument provides comprehensive criteria for assessing the quality of written health information. This dual-assessment approach was crucial for uncovering insights into the credibility and educational value of the health information being disseminated by the selected websites during a critical period of the pandemic. Multimedia Appendix 1 shows all the criteria of the DISCERN and JAMA benchmarks.

### JAMA Benchmarks

Transparency involves disclosing details about how information is produced, which can affect informed decision-making. We assessed this using the JAMA benchmarks, which evaluate 4 key quality criteria: authorship, attribution, disclosure, and currency. Each website was scored based on how many of these criteria were met, resulting in a total score of 0-4. (refer to Multimedia Appendix 1)

### DISCERN Instrument

The DISCERN tool includes 3 sections: the first two evaluate the reliability and quality of the information provided in text form while the third assesses the overall quality of the publication. Questions are rated on a scale from 1 (clearly no) to 5 (clearly yes), with scores of 2 and 4 allocated for criteria that are met to a partial extent. To calculate the DISCERN scores, a Microsoft Excel sheet was created, dedicating 1 row for each question. Websites were analyzed individually, and scores for each question were manually entered into the respective cells on a scale from 1 to 5. For question 16, the mode function was applied to the responses from questions 1 to 15. The total DISCERN score for a website was determined by summing the scores from rows 1 to 16, represented in the 17th row as the total score. The highest possible total score is 80 and the lowest is 16, with websites being categorized based on their score as high (65 points or above), moderate (between 33 and 64 points), or low (between 16 and 32 points) in quality (refer to Multimedia Appendix 1)

### Piloting the Study

Before evaluating the websites, the study tools were explained to the 2 investigators, and they were taught how to use and score them. They identified and addressed any difficulties and disagreements. Then, they independently assessed 10 websites on different topics and resolved any disagreements.

### Analyses

Besides analyzing JAMA and DISCERN scores for each website, we compared them across various categories. Since the data were not normally distributed, we used the Kruskal-Wallis test for the analysis. Post hoc comparisons for the Kruskal-Wallis test were performed using the Dunn test with Bonferroni correction. All statistical analyses were conducted using Python, with significance set at *P*<.05.

## Results

### Descriptive Statistics

The categorization of 100 websites based on their content’s exclusivity, affiliation, and type reveals varied distributions. A significant majority, 89% (89/100) of the sites, are partly exclusive to COVID-19-related content, while 11% (11/100) of the sites are completely dedicated to it. In terms of affiliation, the majority, 69% (69/100) of the sites, are aligned with news organizations, followed by 11% (11/100) of the sites that are commercially affiliated, 8% (8/100) of the sites with government bodies, 6% (6/100) of the sites with medical centers, 4% (4/100) of the sites with nonprofit organizations, and 2% (2/100) of the sites with universities. As for the type of content they provide, 66% (66/100) of the sites predominantly feature human interest stories, 30% (30/100) of the sites focus on providing medical facts, and 4% (4/100) of the sites offer question-and-answer formats.

### JAMA Benchmarks

**Figure 1.**
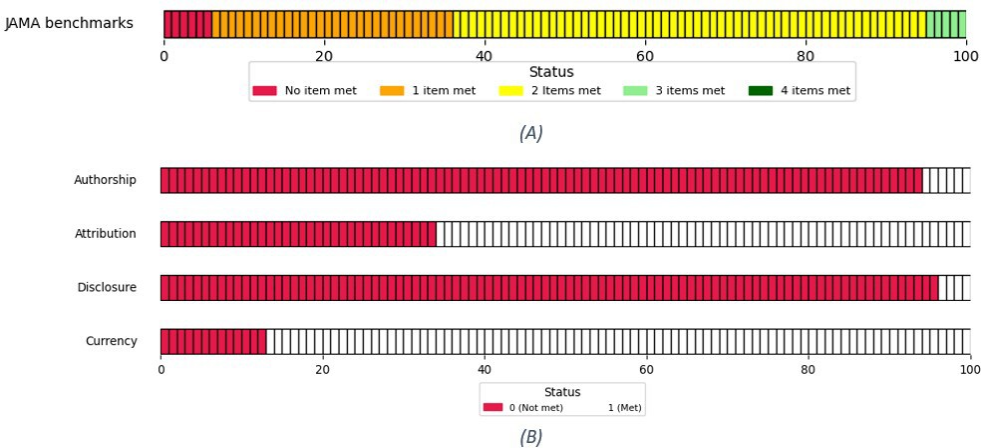
Distributions of JAMA benchmarks and 4 criteria. (A) Distribution of JAMA benchmark score (noncumulative). (B) Distribution of each JAMA quality criteria. JAMA: Journal of the American Medical Association.

Our JAMA benchmarks analyses showed that none of the websites met all 4 items, 5% (5/100) of the websites met 3 items, 59% (59/100) of the websites met 2 items, 30% (30/100) of the websites met only 1 item, and 6% (6/100) of the websites met no JAMA items. However, the absence of any website that met all 4 benchmarks highlights a significant gap in achieving the highest compliance standard set by JAMA. The distribution of websites based on the number of items met is shown in Figure 1A. On average, all websites achieved a mean JAMA score of 1.63 (SD 0.675). Figure 1B illustrates the percentage of websites meeting each of the 4 JAMA benchmark criteria: authorship, attribution, disclosure, and currency. A vast majority of websites failed to meet the authorship (94/100, 94%) and disclosure (96/100, 96%) criteria, indicating significant gaps in transparency and quality due to the lack of proper authorship and disclosure. These results highlight the overall unsatisfactory and low quality of the information provided by most websites. In addition, a moderate proportion of websites did not meet the attribution criterion (34/100, 34%) and currency criterion (13/100, 13%), underscoring the need for improved adherence to these standards.

The JAMA benchmark scores (Table 1) categorize websites based on their exclusivity, content type, and affiliation, displaying the number of criteria met by each group. Notably, no website met all 4 JAMA benchmark criteria, and only 5 websites met 3 items. Surprisingly, no medical center websites met 3 items or more, and no websites with medical facts content met 3 items or more. In addition, only up to 2 items were met.

**Table 1.** Distribution of websites meeting JAMA (Journal of the American Medical Association) benchmark criteria by exclusivity, content type, and affiliation.

Categories	No item met	1 item met	2 items met	3 items met	4 items met	Total
Exclusivity						
Exclusive	4	3	4	0	0	11
Partly exclusive	2	27	55	5	0	89
Content type						
Human interest stories	1	19	41	5	0	66
Medical facts	5	10	15	0	0	30
Questions and answers	0	1	3	0	0	4
Affiliation						
Commercial	1	5	5	0	0	11
Government	5	3	0	0	0	8
Medical center	0	2	4	0	0	6
News	0	18	46	5	0	69
Nonprofit organization	0	2	2	0	0	4
University	0	0	2	0	0	2

For example, among the JAMA benchmark results, 5 news-affiliated websites met 3 benchmark items, indicating higher quality, whereas 5 government-affiliated websites met none of the items, reflecting lower quality. These examples are presented in Multimedia Appendix 2.

Table 2 presents the JAMA and DISCERN scores, including mean (SD) and median, across different website types, content types, and affiliations. The statistical analysis using the Dunn Test with Bonferroni correction, with a significance level set at *P<*.05, reveals significant differences in JAMA scores across various categories. Our analysis demonstrates that all websites scored less than or equal to 2 in transparency, with news websites achieving the mean JAMA score of 1.81 (SD 0.55) and government websites achieving the mean JAMA score of 0.38 (SD 0.52). To our surprise, the transparency (JAMA score) of the analyzed government and medical center–affiliated websites was lower than the news-affiliated websites.

**Table 2.** Analysis of JAMA (Journal of the American Medical Association) benchmarks and DISCERN scores.

Categories	JAMA score, mean (SD)	JAMA score, median (IQR)	DISCERN score, mean (SD)	DISCERN score, median (IQR)
Exclusivity				
Exclusive	1.00 (0.89)	1.0 (0.0-2.0)	64.36 (20.55)	73 (56–79)
Partly exclusive	1.71 (0.61)	2.0 (1.0-2.0)	28.84 (10.78)	25 (25–28)
Content type				
Human interest stories	1.76 (0.61)	2.0 (1.0-2.0)	26.32 (6.77)	25 (25–26)
Medical facts	1.33 (0.76)	1.5 (1.0-2.0)	46.57 (22.50)	36 (25–72)
Questions and answers	1.75 (0.50)	2.0 (1.8-2.0)	35.25 (14.75)	32 (24-41)
Affiliation				
Commercial	1.36 (0.67)	1.0 (1.0-2.0)	34.18 (17.84)	25 (25–34)
News	1.81 (0.55)	2.0 (1.0-2.0)	27.20 (6.45)	25 (25–27)
University	2.00 (0.00)	2.0 (2.0-2.0)	50.00 (31.11)	50 (39–61)

### DISCERN Instrument

**Figure 2.**
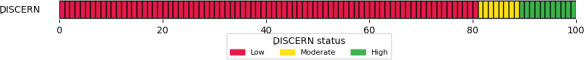
Distribution of DISCERN score (noncumulative).

Figure 2 displays the evaluation of 100 websites based on the DISCERN instrument, categorized into 3 levels according to their achieved score: low (16-32 points), moderate (33-64 points), and high quality (65 points or above). The majority of the analyzed websites, 81% (81/100) of the websites, were of low quality according to the DISCERN criteria, 8% (8/100) of the websites were of moderate quality, and only a small portion, 11% (11/100) of the websites, met the high-quality standards. This highlights the urgent need to improve the quality of online health information, as most websites scored low on quality standards. On average, all websites achieved a mean DISCERN score of 32.75 (SD 16.45). In terms of reliability, the mean score is 20.69 (SD 7.05), and for quality of information questions, the mean score is 10.50 (SD 8.79).

Based on their score, the DISCERN scores in Table 3 categorize websites by their exclusivity, content type, and affiliation into low, moderate, and high quality. Considering the website content type, the ones providing human interest stories (64/100, 64% of our sample) scored low on health quality. Furthermore, it is notable that half of the websites providing medical facts (15/100, 15% of all websites) achieved low quality, despite being expected to be more reliable. No website with questions and answers content type received a high DISCERN score, indicating a need for improvement in information-providing websites. Although the number of university websites is small, it is surprising that 50% (1/2) of university-affiliated websites scored low while universities are expected to meet high academic standards. This assessment underscores a concerning lack of quality and reliability in health information, with a notable trend toward low quality in widely read categories, such as human-interest stories, news, and university-affiliated websites.

**Table 3.** Distribution of websites meeting DISCERN scores criteria by exclusivity, content type, and affiliation.

Categories	Low	Moderate	High	Total
Exclusivity				
Exclusive	2	1	8	11
Partly exclusive	79	7	3	89
Content type				
Human interest stories	64	1	1	66
Medical facts	15	5	10	30
Questions and answers	2	2	0	4
Affiliation				
Commercial	8	2	1	11
Government	3	0	5	8
Medical center	1	1	4	6
News	64	5	0	69
Nonprofit organization	4	0	0	4
University	1	0	1	2

The statistical analysis using the Dunn test with Bonferroni correction, set at a significance level of .05, revealed significant differences in DISCERN scores across various categories during the comparisons. As the DISCERN scores’ mean (SD) and median are shown in Table 2, our analysis indicates that all affiliations have low to moderate DISCERN scores, which are detailed in Multimedia Appendix 3 (mean, SD, and median of reliability and quality of information by categories). In terms of affiliation, medical centers have the highest DISCERN score (mean 61, SD 19.11), while nonprofit organizations and news have the lowest (mean 24, SD 2 and mean 27.2, SD 6.45, respectively) and tend to be low-qualified in both reliability and quality.

## Discussion

### Principal Findings

This study assessed the quality of health information of websites that were circulated the most on Twitter during the early stages of a public health challenge, when the increased use of social media and the accompanying anxiety, fear, and human tendency to seek out information drive individuals to social media platforms like Twitter. Using the established tools to evaluate the quality of health information of JAMA benchmarks and DISCERN, the study evaluated the transparency, reliability, and content quality of those websites. The analysis shows a concerning pattern of achieving an overall low DISCERN score (mean DISCERN score of 32.75, SD 16.45), pointing to a widespread issue of poor health information online. Similarly, our examination using the JAMA benchmarks revealed that these sites were generally of poor quality in terms of transparency, as 95% (95/100) of them at most only met 2 out of 4 criteria of JAMA (see Multimedia Appendix 2). These findings emphasize the urgent need for substantial improvements in presenting and verifying online health information, as the overall quality is unsatisfactory and affects its reliability.

Regarding JAMA benchmarks, while the most significant shortcomings were found in disclosure and authorship, our study also uncovered that several websites lacked proper currency and attribution. These elements are crucial for maintaining the relevance and credibility of health-related information. Promoting transparency, establishing credibility, ensuring the information is up to date, and allowing for source verification are essential practices that should be standard across all health-related websites according to JAMA benchmarks.

Regarding DISCERN and considering the websites’ affiliation, the average DISCERN score of each affiliation was low to moderate. It is worth noting that while in our analyzed websites, we have seen government and medical center websites that achieved high DISCERN scores, the overall performance of these types of websites is also considered moderate. This indicates that only some websites provided information that could be considered somewhat reliable, highlighting that there is significant room for improvement to ensure the health information provided meets both reliability and quality criteria.

The area where medical center websites fall short in the reliability section of DISCERN is related to addressing uncertainty, while government websites fall short in addressing balanced and unbiased information as well as uncertainty. It should be noted that other websites, which represent a significant portion of all, lacked clear aims, failed to achieve those aims, and demonstrated poor relevance, clarity of sources, and currency in terms of reliability. Moreover, these websites struggled with addressing uncertainty and providing balanced and unbiased information, further affecting their overall reliability. Specifically, in the second section of DISCERN, medical center websites underperformed in areas related to treatment and prevention, such as explaining treatment processes, risks and benefits, the consequences of not treating, impact on quality of life, availability of options, and decision-making support. These gaps highlight urgent areas for improvement to enhance patient understanding and decision-making capabilities. These sites also underperformed in key areas of information quality, such as explaining treatment options, outlining potential outcomes, and supporting decision-making processes.

Our study is not without limitations. This study faces several methodological constraints that should be acknowledged. Our analysis was restricted to English-language tweets from March 2020, which limits our ability to capture information-sharing patterns within non-English-speaking communities and may not reflect evolving communication behaviors throughout the pandemic. The Twitter application programming interface’s data-sharing policies and technical restrictions meant we could only rehydrate 28.4 million tweets from the original dataset, with additional constraints from rate limits and the unavailability of tweets that users subsequently deleted or made private. Our methodology of prioritizing the top 100 tweets with URLs, while useful for identifying widely circulated content, may have introduced a bias in that less shared sources, such as government health portals. The exclusion criteria we applied—removing non-English sites, statistical dashboards, video content, and subscription-based sources—though necessary for analytical consistency, further reduced the comprehensiveness of information sources we could examine. Furthermore, we only had 2 university-affiliated websites in our sample. University-affiliated websites showed moderate levels of information quality according to the DISCERN criteria. This makes it challenging to draw comprehensive conclusions about their overall performance. In addition, we acknowledge that the JAMA and DISCERN instruments evaluate only the quality of website content and do not capture user interaction dynamics, such as tweet framing, images, or retweet patterns. Our focus was therefore limited to assessing the source quality of linked health information, rather than the behavioral influence of how this information was shared on Twitter.

These findings highlight the urgent need to continuously assess and improve online health information. Ensuring that health websites meet high standards of transparency, reliability, and quality is crucial, especially during public health emergencies. This emphasizes the importance of addressing these issues through targeted recommendations to enhance the credibility and reliability of online health information during a public health crisis. Without consistent oversight and improvement, we risk repeating the same shortcomings in future, potentially more challenging situations. Implementing targeted enhancements in editorial standards, transparency practices, and the presentation of health information, along with public education, is crucial to ensure that reliable and credible content is available, especially during public health emergencies. Continuous evaluations are essential to prepare for future crises, where the quality of information will play a key role in shaping public health responses and maintaining public trust.

### Implications

The implications of our research for enhancing the overall quality and reliability of the health information provided during the early stages of a health crisis are as follows.

Considering DISCERN, websites affiliated with medical centers, government agencies, and universities generally have higher scores for quality and reliability but still fall into the moderate range. Enhancing quality and reliability involves ensuring detailed and clear information, such as comprehensive descriptions of how each treatment works, the benefits and risks, the consequences of not using treatments, the impact on quality of life, the availability of multiple treatment options, and support for shared decision-making. In addition, implementing regular content updates to ensure information remains current is essential, with dedicated teams assigned to review and update content periodically.

News and commercial websites, although very popular, often provide lower-quality and less reliable information. To improve their DISCERN scores, these websites should implement rigorous editorial standards and quality control measures to ensure accurate, reliable, and unbiased health information. Employing fact-checkers and medical experts to review and verify health content before publication is crucial. Training content creators on best practices for creating high-quality health information, including proper citation of sources and avoidance of sensationalism, will further enhance the quality.

Considering JAMA, websites need to address all criteria, including currency, authorship, attribution, and disclosure, to improve their overall scores. This involves ensuring that health information is up-to-date, clearly authored by qualified experts, properly attributed to credible sources, and transparently disclosed regarding any potential conflicts of interest.

To ensure high-quality health information on platforms like Twitter during a public health emergency, it is crucial to educate the public on identifying credible sources through community programs in schools and workplaces. Social media should enforce strict controls, include health expert reviews, and promote verified content. Clear authorship and easy source access are necessary. Collaborations with health organizations will ensure the dissemination of reliable information, enhancing informed decision-making and public trust. These improvements will boost the overall quality and reliability of health information.

Beyond specific technical and editorial improvements, our study addresses broader and persistent challenges in the health information shared on social media platforms. Rather than offering a retrospective critique of a past crisis, the research highlights systemic issues in online health communication that extend far beyond the COVID-19 context. By applying established quality assessment tools, such as DISCERN and JAMA, we uncovered widespread structural deficiencies—such as the lack of authorship transparency, source attribution, and evidence-based reliability—in widely circulated content on social media. These shortcomings are not crisis-specific. They reveal fundamental vulnerabilities in how health information is produced and disseminated across social platforms. The early pandemic period served as a natural experiment that exposed these weaknesses, underscoring the need for proactive preparedness in future public health emergencies. The principles of transparency, accuracy, and proper attribution must be upheld consistently, regardless of the type of crisis or the platform involved. Moreover, the insights drawn from this study hold enduring relevance, as improving the quality of health information is an ongoing priority that directly influences public trust, informed decision-making, and effective crisis response.

Building on these broader insights, our findings underscore the importance of actionable intelligence for stakeholders. Public health organizations can implement these insights by developing and disseminating preapproved messaging templates to facilitate rapid and effective crisis communication and by establishing real-time monitoring systems to detect and counter false information trends. Social media platforms are encouraged to adjust their algorithms to prioritize verified health content and to promote centralized, in-platform health information hubs that offer timely and reliable updates developed in partnership with public health experts. Policymakers can support these efforts by enacting regulations that mandate transparency, authorship disclosure, and attribution in digital health content, while also introducing quality certification frameworks that help users recognize trusted sources. They should further consider measures to ensure algorithmic accountability, particularly when it comes to the amplification of misleading health information. Meanwhile, health communicators must integrate structured quality evaluation tools, such as those based on the JAMA and DISCERN frameworks, into their content development workflows. Providing training in health literacy, ethical communication, and false information detection to communication teams will ensure that messages remain trustworthy and effective across diverse audiences. By adopting these cross-sector strategies, stakeholders can collaboratively strengthen the digital health information environment and enhance preparedness for future public health emergencies.

In summary, this study not only reveals critical shortcomings in the quality of health information shared on social media during a public health crisis but also offers a practical roadmap for improving future communication strategies. By addressing both systemic and actionable gaps, our findings contribute to a more resilient digital health ecosystem—one that supports public trust, informed choices, and effective crisis response.

### Conclusion

This study reveals a significant issue with the quality, reliability, and transparency of online health-related information, particularly evident during the early stages of a health emergency when social media becomes a primary platform for the dissemination of information. The importance of such evaluations becomes even more pronounced during crises like the COVID-19 pandemic, where access to reliable and high-quality information can significantly impact public health outcomes. The websites analyzed exhibited low quality, with an average DISCERN score of 32.75 (SD 16.45), highlighting widespread deficiencies in health information. Similarly, our analysis using JAMA benchmarks demonstrated generally low transparency, with 95% (95/100) of websites achieving at most 2 JAMA criteria, emphasizing the need for drastic improvements. The extensive shortcomings observed across frequently shared websites on Twitter underscore the critical need for continuous evaluation and improvement of online health content.

## Supplementary material

10.2196/70756 Multimedia Appendix 1 Introduction to DISCERN and JAMA (Journal of the American Medical Association) benchmark criteria.

10.2196/70756 Multimedia Appendix 2 Quality health information analysis of the 100 websites most frequently retweeted on Twitter in March 2020 during the COVID-19 pandemic.

10.2196/70756 Multimedia Appendix 3 Analysis of DISCERN scores, reliability, and quality of information by website exclusivity, content type, and affiliation (reliability range: 8-40 and quality of information range: 7-35).

## ABBREVIATIONS

JAMA: Journal of the American Medical Association
