# Supplementary material for: Quality Assessment of Health Information on Social Media During a Public Health Crisis: Infodemiology Study
Source: JMIR Infodemiology. 2025 Oct 24;5:e70756. doi: 10.2196/70756 (PMC12551971; doi:10.2196/70756)
Supplement: Multimedia Appendix 1 [file infodemiology-v5-e70756-s001.docx]

Introduction to DISCERN and JAMA Benchmark Criteria

| **Discern** | **JAMA Benchmark** |
| --- | --- |
| **Reliability**: 1. Are the aims clear?  2. Does it achieve its aims?  3. Is it relevant?  4. Is it clear what sources of information were used to compile the publication (other than the author or producer)?  5. Is it clear when the information used or reported in the publication was produced?  6. Is it balanced and unbiased?  7. Does it provide details of additional sources of support and information?  8. Does it refer to areas of uncertainty?  **Quality of Information:**  9. Does it describe how each treatment works?  10. Does it describe the benefits of each treatment?  11. Does it describe the risks of each treatment?  12. Does it describe what would happen if no treatment is used?  13. Does it describe how the treatment choices affect the overall quality of life?  14. Is it clear that there may be more than one possible treatment choice?  15. Does it provide support for shared decision-making?  **Overall:**  16. Based on the answers to all of the above questions, rate the overall quality of the publication as a source of information about treatment choices | 1. Authorship  -List authors, contributors, their affiliations, and relevant credentials  2. Attribution -Clearly list all references and sources for the content.  -Note relevant copyright information.  3. Disclosure -Fully and prominently disclose the website's "ownership," as well as any sponsorships, advertising, underwriting, and commercial funding.  4. Currency -Indicate the dates when the content was posted and updated. |
