# Supplementary material for: Quality Assessment of Health Information on Social Media During a Public Health Crisis: Infodemiology Study
Source: JMIR Infodemiology. 2025 Oct 24;5:e70756. doi: 10.2196/70756 (PMC12551971; doi:10.2196/70756)
Supplement: Multimedia Appendix 2 [file infodemiology-v5-e70756-s002.docx]

Quality Health Information Analysis of the 100 Websites Most Frequently Retweeted on Twitter in March 2020 During the COVID-19 Pandemic

| **Row** | **Website URL** | **Exclusivity** | **Content** | **Affiliation** | **JAMA-Score** | **DISCERN-Score** | **DISCERN Categorized score** |
| --- | --- | --- | --- | --- | --- | --- | --- |
| 1 | <https://www.vox.com/2020/3/10/21171481/coronavirus-us-cases-quarantine-cancellation> | Partly exclusive | questions and answers | News | 2 | 55 | Moderate |
| 2 | <https://bnonews.com/index.php/2020/02/the-latest-coronavirus-cases/> | Partly exclusive | medical facts | News | 1 | 28 | Low |
| 3 | <https://www.imperial.ac.uk/media/imperial-college/medicine/sph/ide/gida-fellowships/Imperial-College-COVID19-NPI-modelling-16-03-2020.pdf> | Partly exclusive | medical facts | University | 2 | 72 | High |
| 4 | <https://www.npr.org/2020/03/19/818192535/burr-recording-sparks-questions-about-private-comments-on-covid-19> | Partly exclusive | human interest stories | News | 3 | 25 | Low |
| 5 | <https://graphics.reuters.com/CHINA-HEALTH-SOUTHKOREA-CLUSTERS/0100B5G33SB/index.html> | Partly exclusive | medical facts | News | 1 | 25 | Low |
| 6 | <https://www.latimes.com/politics/story/2020-03-13/trump-administration-blocks-states-use-medicaid-respond-coronavirus-crisis> | Partly exclusive | human interest stories | News | 2 | 29 | Low |
| 7 | <https://medium.com/@tomaspueyo/coronavirus-act-today-or-people-will-die-f4d3d9cd99ca> | Partly exclusive | questions and answers | Commercial | 2 | 38 | Moderate |
| 8 | <http://4.nbcny.com/nEX9Y2K> | Partly exclusive | human interest stories | News | 1 | 25 | Low |
| 9 | <https://trib.al/7iDSrT0> | Partly exclusive | human interest stories | News | 1 | 25 | Low |
| 10 | <https://www.3dprintingmedia.network/covid-19-3d-printed-valve-for-reanimation-device/> | Partly exclusive | human interest stories | Commercial | 2 | 29 | Low |
| 11 | <https://time.com/5806312/coronavirus-treatment-cost/> | Partly exclusive | human interest stories | News | 2 | 25 | Low |
| 12 | <https://bit.ly/39q4BnF> | Partly exclusive | human interest stories | News | 2 | 25 | Low |
| 13 | <https://www.cnn.com/2020/03/13/us/nba-player-donations-coronavirus-trnd/index.html> | Partly exclusive | human interest stories | News | 2 | 25 | Low |
| 14 | <https://theintercept.com/2020/03/12/matt-gaetz-florida-paid-sick-leave-coronavirus/> | Partly exclusive | human interest stories | Nonprofit Organization | 2 | 25 | Low |
| 15 | <http://nhs.uk/coronavirus> | Partly exclusive | human interest stories | Commercial | 0 | 76 | High |
| 16 | <https://blog.moneysavingexpert.com/2020/03/i-m-making-p1m-available-to-fund-urgent-small-charity-coronaviru/> | Partly exclusive | human interest stories | Commercial | 1 | 25 | Low |
| 17 | <https://www.who.int/emergencies/diseases/novel-coronavirus-2019/advice-for-public> | Exclusive | medical facts | Medical Center | 2 | 72 | High |
| 18 | <https://www.newsweek.com/prophet-prayer-muhammad-covid-19-coronavirus-1492798?amp=1> | Partly exclusive | human interest stories | News | 1 | 22 | Low |
| 19 | <http://bit.ly/337yabc> | Partly exclusive | medical facts | News | 2 | 60 | Moderate |
| 20 | <https://www.usatoday.com/story/opinion/2020/03/15/coronavirus-stay-home-hel-america-save-lives-column/5054241002/> | Partly exclusive | human interest stories | News | 2 | 26 | Low |
| 21 | <https://www.buzzfeed.com/alexwickham/coronavirus-uk-strategy-deaths> | Partly exclusive | human interest stories | News | 1 | 28 | Low |
| 22 | <https://www.independent.co.uk/news/world/americas/us-politics/coronavirus-video-trump-pandemic-team-cut-2018-a9405191.html> | Partly exclusive | human interest stories | News | 1 | 32 | Low |
| 23 | <https://sunnybrook.ca/research/media/item.asp?c=2&i=2069&f=covid-19-isolated-2020> | Exclusive | medical facts | Medical Center | 2 | 31 | Low |
| 24 | <https://www.theguardian.com/world/2020/mar/16/no-news-is-good-news-big-brother-germany-cut-off-from-covid-19> | Partly exclusive | human interest stories | News | 1 | 18 | Low |
| 25 | <http://www.who.int/COVID-19> | Exclusive | medical facts | Medical Center | 1 | 77 | High |
| 26 | <https://www.rawstory.com/2020/03/gop-blocking-coronavirus-bill-because-it-limits-how-much-drugmakers-can-charge-for-a-vaccine-report/> | Partly exclusive | human interest stories | News | 2 | 25 | Low |
| 27 | <https://www.wired.com/story/coronavirus-donald-trump-google-website/> | Partly exclusive | human interest stories | News | 2 | 32 | Low |
| 28 | <https://www.independent.co.uk/news/health/coronavirus-conference-new-york-cancelled-council-foreign-relations-business-a9393371.html?utm_medium=Social&utm_source=Twitter#Echobox=1583923126> | Partly exclusive | human interest stories | News | 1 | 27 | Low |
| 29 | <https://www.ft.com/content/a26fbf7e-48f8-11ea-aeb3-955839e06441> | Partly exclusive | human interest stories | News | 3 | 30 | Low |
| 30 | <https://cbsn.ws/33sb67i> | Partly exclusive | human interest stories | News | 2 | 25 | Low |
| 31 | <https://www.smithsonianmag.com/history/journal-plague-year-180965222/> | Partly exclusive | medical facts | Nonprofit Organization | 2 | 25 | Low |
| 32 | <http://apne.ws/Zm9QjND> | Partly exclusive | medical facts | News | 1 | 25 | Low |
| 33 | <https://www.huffpost.com/entry/senate-gop-blocks-emergency-paid-sick-leave-legislation-from-moving-forward_n_5e691016c5b68d61645ebff9> | Partly exclusive | human interest stories | News | 2 | 25 | Low |
| 34 | <https://www.bostonglobe.com/2020/03/13/opinion/coronavirus-cautionary-tale-italy-dont-do-what-we-did/> | Partly exclusive | human interest stories | News | 2 | 25 | Low |
| 35 | <http://bbc.in/38NGApD> | Partly exclusive | human interest stories | News | 1 | 29 | Low |
| 36 | <https://www.cnbc.com/2020/03/15/coronavirus-germany-tries-to-stop-us-luring-away-firm-seeking-vaccine.html> | Partly exclusive | medical facts | News | 2 | 32 | Low |
| 37 | <https://www.prnewswire.com/news-releases/oscar-launches-first-testing-center-locator-for-covid-19-301023288.html> | Partly exclusive | medical facts | Commercial | 2 | 28 | Low |
| 38 | <https://bit.ly/38RaaKV> | Partly exclusive | human interest stories | News | 2 | 26 | Low |
| 39 | <https://www.politico.com/news/2020/03/07/trump-coronavirus-management-style-123465> | Partly exclusive | human interest stories | News | 2 | 26 | Low |
| 40 | <http://www.rfa.org/english/news/china/wuhan-deaths-03272020182846.html> | Partly exclusive | human interest stories | Nonprofit Organization | 1 | 25 | Low |
| 41 | <https://osf.io/fd4rh/?view_only=c2f00dfe3677493faa421fc2ea38e295> | Partly exclusive | medical facts | University | 2 | 28 | Low |
| 42 | <https://www.news.com.au/lifestyle/health/health-problems/chinese-doctors-say-coronavirus-like-a-combination-of-sars-and-aids-can-cause-irreversible-lung-damage/news-story/f58f19c5eeae99b845c54e2d2b9305ca> | Partly exclusive | medical facts | News | 2 | 40 | Moderate |
| 43 | <https://www.politico.com/news/2020/03/06/coronavirus-testing-failure-123166> | Partly exclusive | medical facts | News | 1 | 25 | Low |
| 44 | <https://www.npr.org/2020/03/02/811352024/all-bushfires-extinguished-in-australias-hardest-hit-new-south-wales-officials-s> | Partly exclusive | human interest stories | News | 3 | 25 | Low |
| 45 | <https://www.politico.com/news/2020/03/13/jared-kushner-combat-coronavirus-facebook-127941> | Partly exclusive | human interest stories | News | 2 | 25 | Low |
| 46 | <https://www.vice.com/en_us/article/93988v/whole-foods-suggests-that-workers-share-paid-time-off-during-coronavirus> | Partly exclusive | human interest stories | Commercial | 2 | 25 | Low |
| 47 | <http://www.businessinsider.com/trump-administration-tried-to-pay-germans-scientists-for-coronavirus-cure-2020-3> | Partly exclusive | human interest stories | News | 2 | 27 | Low |
| 48 | <https://www.cnn.com/2020/03/14/health/coronavirus-asymptomatic-spread/index.html> | Partly exclusive | medical facts | News | 2 | 42 | Moderate |
| 49 | <https://medium.com/@Cancerwarrior/covid-19-why-we-should-all-wear-masks-there-is-new-scientific-rationale-280e08ceee71> | Partly exclusive | medical facts | Commercial | 1 | 61 | Moderate |
| 50 | <https://cnn.it/398M5A9> | Partly exclusive | human interest stories | News | 2 | 25 | Low |
| 51 | <https://www.esquire.com/news-politics/politics/a31755559/coronavirus-8-republican-senators-vote-against-relief-package/> | Partly exclusive | human interest stories | Commercial | 1 | 21 | Low |
| 52 | <https://trib.al/5QzbHfo> | Partly exclusive | human interest stories | News | 2 | 25 | Low |
| 53 | <https://www.nhs.uk/conditions/coronavirus-covid-19/> | Partly exclusive | medical facts | Government | 0 | 70 | High |
| 54 | [http://coronavirus.gov](http://coronavirus.gov/) | Exclusive | medical facts | Government | 0 | 79 | High |
| 55 | <https://www.politico.eu/article/germany-confirms-that-donald-trump-tried-to-buy-firm-working-on-coronavirus-vaccine/> | Partly exclusive | human interest stories | News | 1 | 25 | Low |
| 56 | <https://www.vanityfair.com/news/2020/03/trump-germaphobe-in-chief-struggles-to-control-the-covid-19-story> | Partly exclusive | human interest stories | News | 1 | 25 | Low |
| 57 | <https://www.politico.com/news/2020/03/12/trump-coronavirus-travel-europe-resorts-126808> | Partly exclusive | human interest stories | News | 2 | 25 | Low |
| 58 | <https://www.theguardian.com/world/2020/mar/18/scientists-say-mass-tests-in-italian-town-have-halted-covid-19?CMP=twt_gu&utm_source=Twitter&utm_medium#Echobox=1584567735> | Partly exclusive | medical facts | News | 2 | 30 | Low |
| 59 | <http://bit.ly/2xDnG7X> | Exclusive | medical facts | Medical Center | 2 | 43 | Moderate |
| 60 | <https://bit.ly/2IHqeUV> | Partly exclusive | human interest stories | News | 1 | 25 | Low |
| 61 | <https://www.npr.org/2020/03/12/814881355/white-house-knew-coronavirus-would-be-a-major-threat-but-response-fell-short> | Partly exclusive | human interest stories | News | 2 | 25 | Low |
| 62 | <https://act.nationalnursesunited.org/page/-/files/graphics/NU-Quarantine-RN-press-conf-statement.pdf> | Partly exclusive | human interest stories | Nonprofit Organization | 1 | 21 | Low |
| 63 | <https://nypost.com/2020/03/12/trump-passes-coronavirus-test-with-flying-colors-goodwin/> | Partly exclusive | human interest stories | News | 2 | 25 | Low |
| 64 | <https://www.mirror.co.uk/news/politics/coronavirus-nhs-steps-up-fight-21694418> | Partly exclusive | human interest stories | News | 2 | 24 | Low |
| 65 | <https://trib.al/tNxd1w6> | Partly exclusive | human interest stories | News | 2 | 26 | Low |
| 66 | <https://abcn.ws/2TVWbzh> | Partly exclusive | human interest stories | News | 2 | 25 | Low |
| 67 | [http://www.coronavirus.gov](http://www.coronavirus.gov/) | Exclusive | medical facts | Government | 0 | 79 | High |
| 68 | <https://www.gov.uk/government/publications/coronavirus-bill-what-it-will-do/what-the-coronavirus-bill-will-do> | Exclusive | medical facts | Government | 1 | 26 | Low |
| 69 | <https://www.nbcnews.com/politics/white-house/mismanagement-missed-opportunities-how-white-house-bungled-coronavirus-response-n1158746> | Partly exclusive | human interest stories | News | 2 | 26 | Low |
| 70 | <https://newsinfo.inquirer.net/1239171/fda-oks-covid-19-test-kits-developed-by-up-scientists> | Partly exclusive | human interest stories | News | 2 | 31 | Low |
| 71 | <https://www.propublica.org/article/the-trump-administration-drove-him-back-to-china-where-he-invented-a-fast-coronavirus-test?utm_source=pardot&utm_medium=email&utm_campaign=majorinvestigations> | Partly exclusive | human interest stories | News | 2 | 25 | Low |
| 72 | <https://www.breitbart.com/politics/2020/03/13/pollak-democrats-pushed-impeachment-while-coronavirus-spread/> | Partly exclusive | human interest stories | News | 2 | 25 | Low |
| 73 | <https://www.bbc.com/news/world-asia-china-51364382> | Partly exclusive | human interest stories | News | 2 | 30 | Low |
| 74 | <https://thehill.com/homenews/media/485147-rep-garamendi-nih-director-fauci-cancelled-on-five-sunday-talk-shows-after> | Partly exclusive | questions and answers | News | 2 | 25 | Low |
| 75 | <https://edd.ca.gov/about_edd/coronavirus-2019.htm> | Partly exclusive | medical facts | Government | 1 | 24 | Low |
| 76 | <https://www.npr.org/2020/03/19/818192535/burr-recording-sparks-questions-about-private-comments-on-covid-19?utm_campaign=storyshare&utm_source=twitter.com&utm_medium=social> | Partly exclusive | human interest stories | News | 3 | 25 | Low |
| 77 | <https://cnn.it/2PxqtFP> | Partly exclusive | human interest stories | News | 2 | 25 | Low |
| 78 | <https://preml.ge/klf8n> | Partly exclusive | human interest stories | Commercial | 1 | 25 | Low |
| 79 | [http://NHS.uk/coronavirus](http://nhs.uk/coronavirus) | Exclusive | medical facts | Medical Center | 1 | 70 | High |
| 80 | <https://www.cdc.gov/coronavirus/2019-ncov/index.html> | Exclusive | medical facts | Government | 0 | 79 | High |
| 81 | <https://www.tampabay.com/florida-politics/buzz/2020/02/28/whats-on-mike-pences-schedule-as-coronavirus-point-man-a-fundraiser-in-florida/> | Partly exclusive | human interest stories | News | 1 | 24 | Low |
| 82 | <https://www.cnn.com/2020/03/12/politics/congress-outrage-testing-covid-19/index.html> | Partly exclusive | human interest stories | News | 2 | 25 | Low |
| 83 | <https://time.com/5809038/coronavirus-flatten-curve/> | Partly exclusive | medical facts | News | 2 | 25 | Low |
| 84 | <https://www.politico.com/news/2020/03/11/trump-emergency-declaration-coronavirus-message-125902> | Partly exclusive | human interest stories | News | 2 | 25 | Low |
| 85 | <https://www.nakedcapitalism.com/2020/03/boeing-crashes-43-billion-in-share-buybacks-turn-into-existential-threat.html> | Partly exclusive | human interest stories | News | 2 | 20 | Low |
| 86 | <https://www.dailymail.co.uk/health/article-8091401/Germany-using-drive-centres-swab-patients-coronavirus.html> | Partly exclusive | human interest stories | News | 1 | 34 | Moderate |
| 87 | <https://daily.bandcamp.com/features/bandcamp-covid-19-fundraiser> | Partly exclusive | human interest stories | Commercial | 2 | 25 | Low |
| 88 | <https://www.who.int/emergencies/diseases/novel-coronavirus-2019/advice-for-public/myth-busters> | Exclusive | medical facts | Medical Center | 2 | 73 | High |
| 89 | <https://www.axios.com/timeline-the-early-days-of-chinas-coronavirus-outbreak-and-cover-up-ee65211a-afb6-4641-97b8-353718a5faab.html?utm_source=twitter&utm_medium=social&utm_campaign=organic&utm_content=1100> | Partly exclusive | medical facts | News | 2 | 25 | Low |
| 90 | <https://www.buzzfeednews.com/article/stephaniemlee/grand-princess-coronavirus-testing> | Partly exclusive | human interest stories | News | 2 | 27 | Low |
| 91 | <https://www.foxnews.com/media/rick-wilson-melania-trump-be-infected-coronavirus> | Partly exclusive | human interest stories | News | 3 | 21 | Low |
| 92 | <https://www.cdc.gov/coronavirus/2019-nCoV/index.html> | Exclusive | medical facts | Government | 0 | 79 | High |
| 93 | <https://elizabethwarren.com/plans/updated-plan-address-coronavirus?source=soc-WB-ew-tw-Rollout-20200312> | Partly exclusive | medical facts | Government | 1 | 24 | Low |
| 94 | <https://www.reddit.com/r/pcmasterrace/comments/fhb5e4/coronavirus_specific_gpu_projects_are_now/> | Partly exclusive | questions and answers | Commercial | 1 | 23 | Low |
| 95 | <https://trib.al/IJKB96x> | Partly exclusive | human interest stories | News | 1 | 28 | Low |
| 96 | <https://www.motherjones.com/coronavirus-updates/2020/03/minnesota-and-vermont-just-classified-grocery-clerks-as-emergency-workers/> | Partly exclusive | human interest stories | News | 2 | 23 | Low |
| 97 | <https://www.huffpost.com/entry/coronavirus-relief-house-passes_n_5e6c4c4bc5b6747ef11d3467> | Partly exclusive | human interest stories | News | 2 | 26 | Low |
| 98 | <https://www.foxnews.com/media/victor-davis-hanson-china-handling-coronavirus> | Partly exclusive | human interest stories | News | 2 | 23 | Low |
| 99 | <https://www.theverge.com/2020/3/13/21179118/google-coronavirus-testing-screening-website-drive-thru-covid-19> | Partly exclusive | human interest stories | News | 2 | 25 | Low |
| 100 | <https://www.vice.com/en_us/article/v74qzb/atandt-suspends-broadband-usage-caps-during-coronavirus-crisis> | Partly exclusive | human interest stories | News | 2 | 25 | Low |
