# Supplementary material for: Quality Assessment of Health Information on Social Media During a Public Health Crisis: Infodemiology Study
Source: JMIR Infodemiology. 2025 Oct 24;5:e70756. doi: 10.2196/70756 (PMC12551971; doi:10.2196/70756)
Supplement: Multimedia Appendix 3 [file infodemiology-v5-e70756-s003.docx]

Analysis of DISCERN Scores, Reliability, and Quality of Information by Website Exclusivity, Content-Type, and Affiliation. (Reliability (range: 8-40) and Quality of information (range: 7-35))

| **Exclusivity** | **Reliability (mean)** | **Reliability (median)** | **Reliability (std)** | **Quality of information (mean)** | **Quality of information (median)** | **Quality of information (std)** |
| --- | --- | --- | --- | --- | --- | --- |
| **Exclusive** | 33 | 34 | 7 | 27 | 35 | 13 |
| **Partly exclusive** | 19 | 17 | 5 | 8 | 7 | 5 |
|  |  |  |  |  |  |  |
|  |  |  |  |  |  |  |
| **Content-Type** | **Reliability (mean)** | **Reliability (median)** | **Reliability (std)** | **Quality of information (mean)** | **Quality of information (median)** | **Quality of information (std)** |
| **Human interest stories** | 18 | 17 | 4 | 7 | 7 | 3 |
| **Medical facts** | 27 | 27 | 9 | 17 | 7 | 13 |
| **Questions and answers** | 23 | 20 | 9 | 11 | 11 | 4 |
|  |  |  |  |  |  |  |
|  |  |  |  |  |  |  |
| **Affiliation** | **Reliability (mean)** | **Reliability (median)** | **Reliability (std)** | **Quality of information (mean)** | **Quality of information (median)** | **Quality of information (std)** |
| **Commercial** | 21 | 17 | 8 | 11 | 7 | 9 |
| **Government** | 30 | 35 | 11 | 25 | 35 | 14 |
| **Medical Center** | 32 | 33 | 5 | 26 | 35 | 14 |
| **News** | 19 | 17 | 4 | 7 | 7 | 2 |
| **Nonprofit Organization** | 16 | 17 | 2 | 7 | 7 | 0 |
| **University** | 30 | 30 | 14 | 17 | 17 | 14 |
